# Malaria innovation: new nets, old challenges

**DOI:** 10.2471/BLT.23.021023

**Published:** 2023-10-01

## Abstract

The release of the first nets using chlorfenapyr is a cause for celebration but raises questions regarding how best to leverage innovation. Gary Humphreys and Lynn Eaton report.

On the face of it, this year’s roll-out of mosquito nets treated with the insecticides chlorfenapyr and alpha-cypermethrin is cause for serious celebration, and no-one is happier about it than Mathias Mondy.

Director of strategy, portfolio and project management at the Innovative Vector Control Consortium (IVCC), a not-for-profit product development partnership (PDP), Mondy and his colleagues have been instrumental in supporting the development, evaluation at scale and market uptake of one such net, known as Interceptor G2 (IG2).

“These are the first nets using a new insecticide to be rolled out at scale in 30 years, and they are going to help overcome growing resistance issues,” Mondy says. The issues he refers to have arisen in relation to a class of synthetic pesticides known as pyrethroids.

Credited with bringing about declines in malaria incidence between 2001 and 2015 estimated to be in the order of 70%, pyrethroids have been the mainstay of vector control efforts since the 1990s. And that is part of the problem.

Dr Jan Kolaczinski, who leads the Vector Control and Insecticide Resistance unit within the World Health Organization (WHO) Global Malaria Programme explains: “Pyrethroid-treated nets and indoor residual spraying imposed a massive selective pressure on mosquito populations that has resulted in the development of pyrethroid resistance in many settings, notably in sub-Saharan Africa.”

Added to malaria-eradication headwinds that include climate change, increased humanitarian emergencies and funding shortfalls, pyrethroid resistance has contributed to progress on malaria incidence flatlining since 2017.

“Because chlorfenapyr attacks mosquitoes in a different way to pyrethroids (disrupting their mitochondria rather than attacking their nervous system), mosquitoes are unlikely to be resistant to both products,” says Kolaczinski.

That combination nets work is borne out by randomized controlled trials (RCTs) that took place in Tanzania (in a trial covering 39 000 households) and a second in Benin (in a trial covering 54 000 households). These trials were funded by Unitaid and the Global Fund to Fight AIDS, Tuberculosis and Malaria (the Global Fund) under the IVCC-led initiative known as the New Nets Project (NNP) and found that the IG2 nets nearly halved malaria incidence compared to pyrethroid-only nets.

Trials to assess the cost-effectiveness of the nets under operational pilot conditions across five sub-Saharan countries (Benin, Burkina Faso, Kenya, Mozambique and Rwanda) were run in parallel with the RCTs between 2019 and 2023.

“Now everyone wants these nets.”

David McGuire

“The countries presented different epidemiological, insecticide-resistance and entomological profiles, and the pilots not only confirmed efficacy but also revealed the challenges faced in the field,” says David McGuire, IVCC’s Director of Access and Market Shaping.

Those challenges include inconsistent use. “People have to choose to use them 365 days a year,” says McGuire. “That doesn’t always happen. When it gets really hot, people often choose to sleep outside, for example.”

Any lingering doubts that government procurement officers might have had about the nets were dispelled when WHO issued a policy recommendation in March 2023, covering pyrethroid-chlorfenapyr nets as well as a conditional recommendation for pyrethroid-pyriproxyfen nets.

“Essentially, the recommendation gave governments confidence about the evidence and stimulated demand for the product,” says McGuire, adding: “Now everyone wants these nets.”

Whether governments will be able to afford them is another matter. According to the Global Fund, the pooled procurement standard reference price for a pyrethroid-only net (including hooks, strings and a bag for the net) in the first half of 2023 was 1.98 United States dollars (US$). This compares to the estimated price of US$ 2.70 for a dual-insecticide net of the same dimensions.

The price difference would have been even higher had it not been for the ‘market shaping’ efforts of several partners, including social finance company MedAccess and the Bill & Melinda Gates Foundation, who supported access to the IG2 nets in over 20 countries by providing a volume guarantee that enabled chemical company BASF, the maker of IG2, to reduce the price procurers pay.

According to Kelsey Barrett, a technical officer working in the Strategy Unit at Unitaid, a complementary co-payment mechanism set up by Unitaid and the Global Fund through the New Nets Project, bridged the remaining price gap during the life of the project, which saw 37 million dual-insecticide nets procured.

In August, the Global Fund announced the launch of a new Revolving Facility to negotiate improved supply terms for global health products for the countries it supports. The mechanism also uses volume guarantees, and the first agreement will be for a pyrethroid-chlorfenapyr net known as Permanent Dual.

But even with such accommodations, roll-out is going to take time. “Given the limited financial resources, the nets can’t be deployed everywhere,” says Barrett. She notes, however, that this may not be a bad thing. “It would be helpful to avoid the mistakes of the past,” she says, referring to the widespread and intensive use of pyrethroid-treated nets in the past which led to emergent resistance.

IVCC’s McGuire agrees. “There needs to be a degree of stewardship,” he says, drawing an analogy with antimicrobial use, where responsible, targeted use is considered essential to retaining treatment efficacy.

For Barrett it is important to consider the new nets in the context of other vector control interventions, rather than seeing them as a silver bullet. “Part of the conversation we need to be having with countries considering using the new nets is ensuring we have a really good grip on what might be the most effective mix of new and existing tools in different settings,” she says.

Several promising new tools are becoming available, ranging from outdoor sugar traps to a new indoor residual spray which was recently prequalified by WHO. There are also major developments on the malaria vaccine front with the mass roll-out of the first malaria vaccine.

Meanwhile, pyrethroid-treated nets continue to work in many countries. India is a case in point. According to Dr Ashwani Kumar, Director of the Indian Council of Medical Research Vector Control Research Centre, Puducherry, India, despite heavy use of pyrethroid-treated nets, notably in Odisha, an eastern Indian state on the Bay of Bengal, starting in 2017, the nets continue to be broadly effective. “At the moment, pyrethroid nets are about 80% effective across India,” he says. “There may come a time when they lose their efficacy, and then, obviously, we will have to look for alternatives.”

“It would be helpful to avoid the mistakes of the past.”

Kelsey Barrett

For the time being, on the insecticide front, those alternatives boil down to chlorfenapyr and pyriproxyfen. “We need more,” says McGuire, noting that it is a long journey from new product to approved public health intervention.

BASF started collaborating on the nets with IVCC in 2011, at which point they had already been working for several years trying to solve challenges that included getting the chemicals to bind to the net well enough to resist repeated washes, while remaining bioavailable to the mosquitoes. The product then underwent years of development, two large trials and further pilot studies.

Does the process have to be so long? According to McGuire this is a question that government representatives often ask. “People have taken note of some of the mechanisms used to compress vaccine trial schedules for COVID-19, such as running animal and phase I human trials in parallel. There is considerable interest in using entomological studies as a proxy to anticipate epidemiological impact, as well as the application of mathematic models which are getting better and are expected to mitigate the need for long and expensive trials.”

There have also been questions about the need to run two large-scale trials, as required to inform the development of a WHO recommendation. “People understand the caution when it comes to vaccine trials, but with insecticides you establish whether the product is safe or not for humans quite early on,” says McGuire. “Cutting out one of the trials would significantly reduce costs and save time.”

However trials are organized, any products proving successful still need to be rolled out, a process that requires market-shaping investments. The tripartite call for action put out on this year’s World Malaria Day, ‘Innovate, Invest and Implement’, reflects the triple challenge faced.

WHO’s Kolaczinski spells it out. “Making progress towards the 2030 malaria incidence target is going to require redoubled global focus on this disease, and increased funding for research and development of new tools and interventions.”

**Figure:**
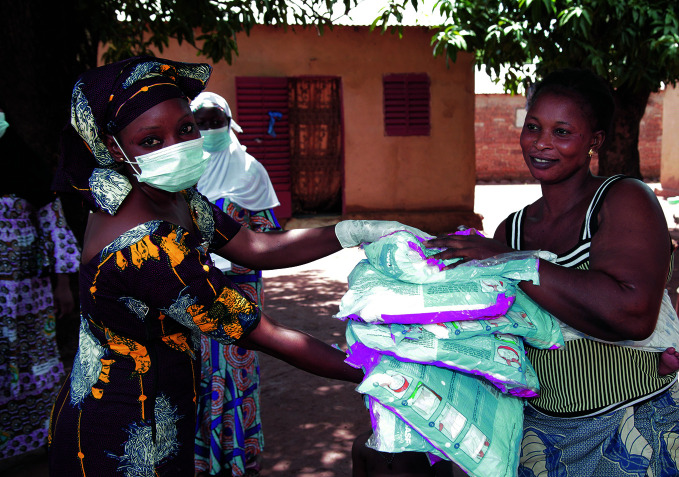
Interceptor G2 nets being distributed during a field trial.

Courtesy of: Population Services International

**Figure:**
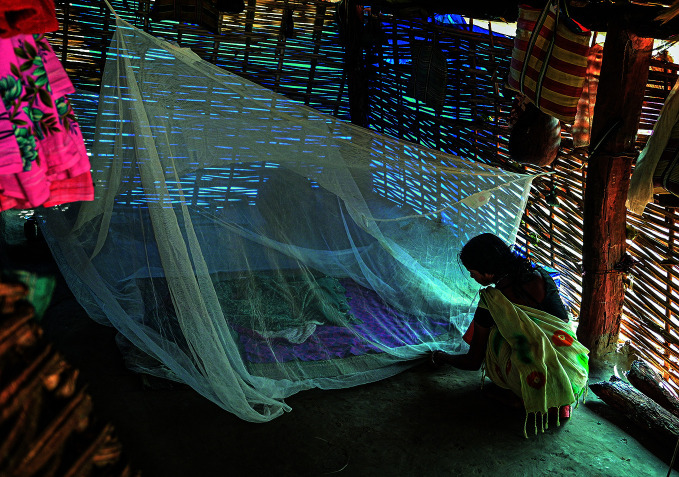
Mother protects her baby with a pyrethroid-treated net in Chhattisgarh, India.

WHO /Atul Loke, Panos Pictures

